# MAPK signaling regulates the efficacy of chemoimmunotherapy

**DOI:** 10.1080/23723556.2022.2054652

**Published:** 2022-04-27

**Authors:** François Ghiringhelli

**Affiliations:** a Leclerc, Equipe Labellisée Ligue Contre le CancerCancer Biology Transfer Platform, Centre Georges-François, Dijon, France; b Centre de Recherche INSERM LNC-UMR1231, Dijon, France; c Univ. Bourgogne Franche-Comté, Dijon, France; d Department of Medical Oncology, Centre Georges-François Leclerc, Dijon, France; e Genetic and Immunology Medical Institute, Dijon, France

**Keywords:** Lung cancer, MEK inhibitor, chemotherapy, immunotherapy, mitophagy, TLR9, immunogenic cell death, CXCL10

## Abstract

Resistance to chemoimmunotherapy is a major issue for cancer care. We recently unravelled the role of mitogen-activated protein kinase (MAPK) to limit the antitumor efficacy of such combination. Inhibitor of MAPK pathway using mitogen-activated protein kinase (MEK) inhibitor in combination with chemotherapy triggers mitophagy of cancer cells, which induces the release of mitochondrial DNA that interact with Toll Like receptor 9 (TLR9) to promote the production of the chemokine CXCL10. CXCL10 could then turn cold tumor into hot tumor, thus leading to improve efficacy of chemoimmunotherapy.

Immunotherapy targeting PD1/PDL1 checkpoint inhibitors changes the field of solid cancer therapy. These treatments become the standard of care in many cancer types. However, resistance is a major issue and many patients fail to gain benefit from such therapy. Many mechanisms could explain the lack of efficacy of checkpoint inhibitors, such as loss of major histocompatibility complex-I expression, a low level of tumor neo-antigens, or poor CD8 infiltration^.[1–3]^

Association of standard of care chemotherapy with checkpoint inhibitor are wildly developed and became first-line standard in many cancer type, like Non-Small Cell Lung Cancer (NSCLC), Small cell Lung Cancer (SCLC), esophageal cancer, gastric cancer, triple negative breast cancer and cervical cancer. The rationale for this association is based on the capacity of chemotherapy to promote immunogenic cell death. This phenomenon involves activation of multiple molecular processes. First, reticulum stress leads to cell surface exposure of calreticulin (CRT)^.[4]^ This “eat me” signal promotes tumor cell phagocytosis by dendritic cells, which are then activated by ATP and high mobility group box 1 release^.[5,6]^ Then, type I Interferon (IFN) release could induce CXCL10 secretion and CD8+ T cell homing in the tumor microenvironment. which promotes immune recruitment and amplifies response to checkpoint inhibitors.

In NSCLC adenocarcinoma, the standard of care involved association of pemetrexed, platin and pembrolizumab.^.[7]^ We developed preclinical models that aimed at modeling immune-resistance: LLC1 model (a model with mutation in *Kras* and *Tp53* gene) or lung tumor induced by the carcinogen urethane (which gives also lung tumors with mutation in *Kras* and *Tp53* gene). These two models are completely resistant to pemetrexed, platin and pembrolizumab association.^.[8]^ Biological analyses underline that chemotherapy in these models induces immunogenic cell death as expected. Despite induction of immunogenic cell death process, chemotherapy is unable to promote CXCL10 production at the tumor site and immune recruitment. Using a drug screening, we observed that drug-inhibiting MAPK pathway enhances CXCL10 production by cancer cells treated by chemotherapy. Importantly, short course of MEK inhibitor, in addition with chemoimmunotherapy, induces *in vivo* CXCL10 production and immune recruitment of CD8 T cells. Many studies underline the potential role of target therapy, especially MEK inhibitors, to mediate immunostimulatory or immunosuppressive effects.^.[9]^ In accordance with our data, short course of MEK and BRAF inhibitors improve efficacy of immunotherapy in melanoma.^[10]^

In a therapeutic point of view, this strategy improves efficacy of chemoimmunotherapy in the two tested experimental models of NSCLCs. Moreover, we observed similar synergy in experimental models of colorectal cancer, triple negative breast cancer and ovarian cancers.

In a mechanistically point of view (Figure 1), we observed that chemotherapy induces radical oxygene species (ROS) production leading to mitochondria damages and MAPK pathway activation. Inhibition of MAPK pathway using MEK inhibitors during chemotherapeutic treatment induces TANK-binding kinase 1 (TBK1) phosphorylation. TBK1 induces the phosphorylation of Optineurin protein, a mitophagy adaptator. Optineurin phosphorylation enhances its binding affinity to ubiquitin chains present on damaged mitochondria. Such phenomenon induces a process of selective mitophagy. In addition, transcriptomic analysis of cancer cells treated with chemotherapy plus MEK inhibitors showed increased *Optn* gene expression during therapy. These data underline the capacity of the combination of MEK inhibitor plus chemotherapy to promote mitophagy process through transcriptional and post traductional effect.

**Figure 1.**
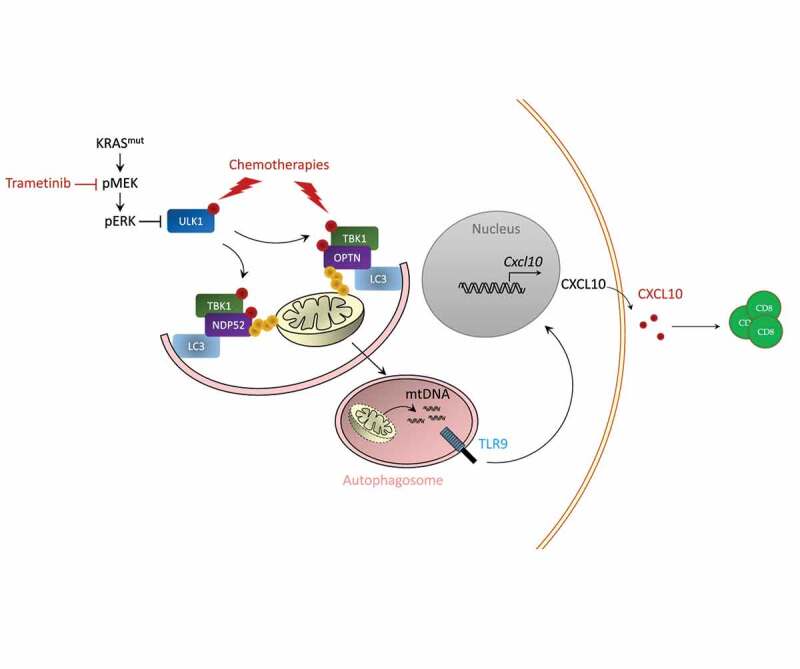
Mechanisms of immunostimulation by MEK inhibition.

Schematic view of the mechanism of action of combination of MEK inhibitors with chemoimmunotherapy. Chemotherapy promotes autophagy and mitophagy by inducing radical oxygen species which activate ULK, OPTN and TKB1. MAPK pathway prevents activation of this pathway by limiting TBK1 and ULK activation. Mitophagy process triggers TLR9 activation and CXCL10 production.

(KRAS: V-Ki-ras2 Kirsten rat sarcoma viral oncogene homolog, MEK: Mitogen-activated protein kinase kinase, ERK: Extracellular signal-regulated kinase, ULK1: Unc-51 like autophagy activating kinase 1, TBK1: TANK-binding kinase 1, OPTN: optineurin, LC3: Microtubule-associated proteins 1A/1B light chain 3B, mtDNA: mitochondrial DNA.)

Electron microscopy analysis confirms that the treatment leads to the generation of mitophagy images, with pictures of mitochondria enclosed in a double membrane. Combination of MEK inhibitors plus chemotherapy also leads to Microtubule-associated proteins 1A/1B light chain 3B (LC3) lipidation and Microtubule-associated proteins 1A/1B light chain 3B (ULK1) phosphorylation; thus, underlining increases in autophagic flux. CRISPR Cas9 invalidation of *Optn* or *Atg5* gene in cancer cells impedes the capacity of the treatment to trigger CXCL10 production by cancer, the recruitment of CD8 T cells and the therapeutic effect of the association.

Electron microscopy analysis confirms that the treatment leads to the generation of mitophagy images, with pictures of mitochondria enclosed in a double membrane. Combination of MEK inhibitors plus chemotherapy also leads to Microtubule-associated proteins 1A/1B light chain 3B (LC3) lipidation and Microtubule-associated proteins 1A/1B light chain 3B (ULK1) phosphorylation; thus, underlining increases in autophagic flux. CRISPR Cas9 invalidation of *Optn* or *Atg5* gene in cancer cells impedes the capacity of the treatment to trigger CXCL10 production by cancer, the recruitment of CD8 T cells and the therapeutic effect of the association.

Electron microscopy analysis confirms that the treatment leads to the generation of mitophagy images, with pictures of mitochondria enclosed in a double membrane. Combination of MEK inhibitors plus chemotherapy also leads to Microtubule-associated proteins 1A/1B light chain 3B (LC3) lipidation and Microtubule-associated proteins 1A/1B light chain 3B (ULK1) phosphorylation; thus, underlining increases in autophagic flux. CRISPR Cas9 invalidation of *Optn* or *Atg5* gene in cancer cells impedes the capacity of the treatment to trigger CXCL10 production by cancer, the recruitment of CD8 T cells and the therapeutic effect of the association.

We could observe that during mitophagy process mitochondrial DNA (mtDNA) is responsible for CXCL10 production. Depletion of mtDNA using ethidum bromide aborts CXCL10 production. Chemotherapy plus MEK inhibitor induces co-localization of the mtDNA-and TLR9 within autophagic vacuoles leading to TLR9 signaling pathway activation. CRISPR Cas9 invalidation of TLR9 impedes CXCL10 production by cancer cells, T cells recruitment and antitumor effect of chemoimmunotherapy plus MEK inhibitor.

In human NSCLC cell lines, we also observed the similar capacity of platin, pemetrexed and MEK inhibitors to promote CXCL10. In NSCLC patients, we observed that expressions of TLR9, Optineurin and CXCL10 are associated with better prognosis in multiple data set of patients treated with checkpoint inhibitors.

These data raise the hypothesis that clinical trial testing the associating short course of MEK inhibition with chemoimmunotherapy will improve efficacy of chemoimmunotherapies, especially in cold tumor or in tumor lacking CXCL10 production. In addition, our data raise the hypothesis that TLR9 agonist could be also interesting to test in combination with chemoimmunotherapies to fight against resistance.

## References

[1] Rizvi NA, Hellmann MD, Snyder A, Kvistborg P, Makarov V, Havel JJ, Lee W, Yuan J, Wong P, Ho TS, et al. Cancer immunology. Mutational landscape determines sensitivity to PD-1 blockade in non-small cell lung cancer. Science. 2015;348(6230):124–3. doi:10.1126/science.aaa1348. 25765070 PMC4993154

[2] Chowell D, Krishna C, Pierini F, Makarov V, Rizvi NA, Kuo F, Morris LGT, Riaz N, Lenz TL, Chan TA. Evolutionary divergence of HLA class I genotype impacts efficacy of cancer immunotherapy. Nat Med. 2019;25(11):1715–1720. doi:10.1038/s41591-019-0639-4. 31700181 PMC7938381

[3] Havel JJ, Chowell D, Chan TA. The evolving landscape of biomarkers for checkpoint inhibitor immunotherapy. Nat Rev Cancer. 2019;19(3):133–150. doi:10.1038/s41568-019-0116-x. 30755690 PMC6705396

[4] Obeid M, Tesniere A, Ghiringhelli F, Fimia GM, Apetoh L, Perfettini J-L, Castedo M, Mignot G, Panaretakis T, Casares N, et al. Calreticulin exposure dictates the immunogenicity of cancer cell death. Nat Med. 2007;13(1):54–61. doi:10.1038/nm1523. 17187072

[5] Apetoh L, Ghiringhelli F, Tesniere A, Obeid M, Ortiz C, Criollo A, Mignot G, Maiuri MC, Ullrich E, Saulnier P, et al. Toll-Like receptor 4-dependent contribution of the immune system to anticancer chemotherapy and radiotherapy. Nat Med. 2007;13(9):1050–1059. doi:10.1038/nm1622. 17704786

[6] Ghiringhelli F, Apetoh L, Tesniere A, Aymeric L, Ma Y, Ortiz C, Vermaelen K, Panaretakis T, Mignot G, Ullrich E, et al. Activation of the NLRP3 inflammasome in dendritic cells induces IL-1beta-dependent adaptive immunity against tumors. Nat Med. 2009;15(10):1170–1178. doi:10.1038/nm.2028. 19767732

[7] Gandhi L, Rodríguez-Abreu D, Gadgeel S, Esteban E, Felip E, De Angelis F, Domine M, Clingan P, Hochmair MJ, Powell SF, et al. Pembrolizumab plus chemotherapy in metastatic non-small-cell lung cancer. N Engl J Med. 2018;378(22):2078–2092. doi:10.1056/NEJMoa1801005. 29658856

[8] Limagne E, Nuttin L, Thibaudin M, Jacquin E, Aucagne R, Bon M, Revy S, Barnestein R, Ballot E, Truntzer C, et al. MEK inhibition overcomes chemoimmunotherapy resistance by inducing CXCL10 in cancer cells. Cancer Cell. 2022 ;40(2):S1535610821006620. doi:10.1016/j.ccell.2021.12.009. 35051357

[9] Petroni G, Buqué A, Zitvogel L, Kroemer G, Galluzzi L. Immunomodulation by targeted anticancer agents. Cancer Cell. 2021;39(3):310–345. doi:10.1016/j.ccell.2020.11.009. 33338426

[10] White MG, Szczepaniak Sloane R, Witt RG, Reuben A, Gaudreau PO, Andrews MC, Feng N, Johnson S, Class CA, Bristow C, et al. Short-Term treatment with multi-drug regimens combining BRAF/MEK-targeted therapy and immunotherapy results in durable responses in Braf-mutated melanoma. Oncoimmunology. 2021;10(1):1992880. doi:10.1080/2162402X.2021.1992880. 34777916 PMC8583008

